# Comment on Blancafort Alias et al. A Multi-Domain Group-Based Intervention to Promote Physical Activity, Healthy Nutrition, and Psychological Wellbeing in Older People with Losses in Intrinsic Capacity: AMICOPE Development Study. *Int. J. Environ. Res. Public Health* 2021, *18*, 5979

**DOI:** 10.3390/ijerph19020860

**Published:** 2022-01-13

**Authors:** Marco Inzitari, Laura Mónica Pérez, Lorena Villa-García, Matteo Cesari

**Affiliations:** 1 REFiT Bcn Research Group, Parc Sanitari Pere Virgili and Vall d’Hebrón Institute of Research (VHIR), 8023 Barcelona, Spain; lperez@perevirgili.cat (L.M.P.); Lorena.v22@gmail.com (L.V.-G.); 2 Faculty of Health Sciences, Universitat Oberta de Catalunya (UOC), 08018 Barcelona, Spain; 3 Doctorate Program, Department of Medicine, Universitat Autònoma de Barcelona, 08035 Barcelona, Spain; 4 QIDA, 08202 Sabadell, Spain; 5 Geriatric Unit, IRCCS Istituti Clinici Scientifici Maugeri, 20138 Milan, Italy; macesari@gmail.com; 6 Faculty of Medicine, Università degli Studi di Milano, 20122 Milan, Italy

We read with interest the article by Blancafort et al. reporting the rationale and process of the “AMICOPE” study [1], which aims to implement a strategy to improve the intrinsic capacity of older adults in Spain. AMICOPE is aligned with the Integrated Care of Older People (ICOPE) strategy proposed by the World Health Organization (WHO) and aims at potentiating the capacities of older adults in the community through the promotion of physical activity, healthy nutritional habits and psychological wellbeing [2]. In particular, we appreciated the idea of following the steps indicated by the Medical Research Council (MRC) to implement a complex intervention, i.e., a multicomponent intervention, which might be highly influenced by contextual factors [3].

This strategy shares many principles and key actions with our +AGIL Barcelona program, which started in 2016 and is still ongoing in one Barcelona’s primary care area (Spain) [4,5]. As in AMICOPE, we followed the same MRC principles to design our intervention, so that +AGIL Barcelona results from the involvement of different stakeholders, including primary care teams (physicians, nurses and social workers), geriatricians, physical therapists, professionals of the third sector, volunteers and end-users. Our program is directed towards older adults with initial frailty (i.e., independent in the basic activities of daily living with the presence of early signs of physical and/or cognitive impairment). The concept of frailty, which can be intended as an alert for a reduction in the physiologic reserve of the older person, is considered a robust prognostic marker of future adverse events. The literature on frailty was determinant in the design of the “positive” concept of intrinsic capacity proposed by the WHO. The two concepts, in fact, share many similarities [6]. Moreover, the methodology and interventions proposed by the WHO for enhancing the intrinsic capacity of older persons represent well-established assets of geriatric medicine, commonly implemented by geriatric teams under a different framework (i.e., management of frailty).

It has been demonstrated that frailty represents an ideal target for preventive interventions against adverse events, especially incident disability. +AGIL Barcelona is based on a community detection strategy by primary healthcare and a comprehensive geriatric assessment conducted by a multidisciplinary team (centered on the role of the geriatrician and the physical therapist). The resulting intervention is tailored to the needs and priorities of the individual and may include up to 10 weekly sessions of adapted physical group exercise complemented by the use of an App for physical activity (ViviFrail©), nutritional recommendations (based on the Mediterranean diet) and specific actions on other different needs (e.g., interventions against cognitive impairment, inadequate sleep, polypharmacy and social isolation).

The +AGIL project intervention strategy is very similar to what was adopted in AMICOPE, although there are some differences in the approach. For example, the design of AMICOPE seems quite selective in the inclusion of participants because it apparently excludes older adults with cognitive impairment or sensorial deficits (conditions that are highly prevalent in older age). +AGIL Barcelona was designed to be more adaptable to the needs of older adults and primary care staff and includes a possibly more heterogeneous population. Interestingly, the preliminary results of +AGIL Barcelona have shown a beneficial impact of the program across different degrees of frailty, suggesting that the intervention is very customizable [5]. On the other hand, AMICOPE seems to have been designed to more specifically act on the promotion of mental and psychological well-being.

The results of the co-creation process, which started from the review of the best available evidence on this topic, represented the basis of +AGIL Barcelona and were published in 2018 [4]. This first manuscript specifically underlined the principles and key elements to implementing such a complex intervention, including the users’ participation and empowerment, the integration of healthcare and community resources, the sustainability overtime and the local dissemination [7]. In 2019, together with the program’s logic model, the preliminary results were published, showing a very high adherence to the program and the positive pre–post impact of the intervention on physical function [5].

The +AGIL Barcelona program is a nationally well-known research-implementation program supported by a national and international dissemination strategy and was also benchmarked with other relevant research in this field conducted at a local level [8]. The program continuously adapts to challenges and opportunities posed by different contexts (such as the COVID-19 pandemic, which pushed the program to reinforce its digital component) and the users’ needs. We believe that +AGIL Barcelona, which is currently implemented, has already shown promising results and accumulated lessons based on its achievements and failures. It might indeed be a valuable learning contribution to similar strategies, such as AMICOPE, which aims to reinforce the intrinsic capacity of older adults in the same setting. It is amenable to working with similar programs (at national and international levels) to develop synergies and collaborative research, for instance, comparing or merging data in the future.

## Data Availability

Not applicable.

## References

[1] Blancafort Alias S., Cuevas-Lara C., Martínez-Velilla N., Zambom-Ferraresi F., Soto M.E., Tavassoli N., Mathieu C., Heras Muxella E., Garibaldi P., Anglada M. (2021). A Multi-Domain Group-Based Intervention to Promote Physical Activity, Healthy Nutrition, and Psychological Wellbeing in Older People with Losses in Intrinsic Capacity: AMICOPE Development Study. Int. J. Environ. Res. Public Health.

[2] World Health Organization (2019). Integrated Care for Older People (ICOPE): Guidance for Person-Centered Assessment and Pathways in Primary Care.

[3] Craig P., Dieppe P., Macintyre S., Michie S., Nazareth I., Petticrew M. (2008). Developing and evaluating complex interventions: The new Medical Research Council guidance. BMJ.

[4] Inzitari M., Pérez L.M., Enfedaque-Montes M.B., Soto L., Díaz F., Gual N., Martin E., Orfila F., Mulero P., Ruiz R. (2018). Integrated primary and geriatric care for frail older adults in the community: Implementation of a complex intervention into real life. Eur. J. Intern. Med..

[5] Pérez L.M., Enfedaque-Montes M.B., Cesari M., Soto-Bagaria L., Gual N., Burbano M.P., Tarazona-Santabalbina F.J., Casas R.M., Díaz F., Martín E. (2019). A Community Program of Integrated Care for Frail Older Adults: +AGIL Barcelona. J. Nutr. Health Aging.

[6] Belloni G., Cesari M. (2019). Frailty and Intrinsic Capacity: Two Distinct but Related Constructs. Front. Med..

[7] Inzitari M., Ruiz D., Martos J., Santaeugenia S. (2016). “Move on against frailty”: Time to raise awareness about frailty and prevention of disability in the community. J. Frailty Aging.

[8] Romera-Liebana L., Orfila F., Segura J.M., Real J., Fabra M.L., Möller M., Lancho S., Ramirez A., Marti N., Cullell M. (2018). Effects of a Primary Care-Based Multifactorial Intervention on Physical and Cognitive Function in Frail, Elderly Individuals: A Randomized Controlled Trial. J. Gerontol. A Biol. Sci. Med. Sci..

